# Acidogenesis, solventogenesis, metabolic stress response and life cycle changes in *Clostridium beijerinckii* NRRL B-598 at the transcriptomic level

**DOI:** 10.1038/s41598-018-37679-0

**Published:** 2019-02-04

**Authors:** Petra Patakova, Barbora Branska, Karel Sedlar, Maryna Vasylkivska, Katerina Jureckova, Jan Kolek, Pavlina Koscova, Ivo Provaznik

**Affiliations:** 1 Department of Biotechnology, University of Chemistry and Technology Prague, Technicka 5, 166 28 Prague, Czech Republic; 2 Department of Biomedical Engineering, Brno University of Technology, Technicka 12, 616 00 Brno, Czech Republic

## Abstract

*Clostridium beijerinckii* NRRL B-598 is a sporulating, butanol and hydrogen producing strain that utilizes carbohydrates by the acetone-butanol-ethanol (ABE) fermentative pathway. The pathway consists of two metabolic phases, acidogenesis and solventogenesis, from which the latter one can be coupled with sporulation. Thorough transcriptomic profiling during a complete life cycle and both metabolic phases completed with flow cytometry, microscopy and a metabolites analysis helped to find out key genes involved in particular cellular events. The description of genes/operons that are closely involved in metabolism or the cell cycle is a necessary condition for metabolic engineering of the strain and will be valuable for all *C*. *beijerinckii* strains and other Clostridial species. The study focused on glucose transport and catabolism, hydrogen formation, metabolic stress response, binary fission, motility/chemotaxis and sporulation, which resulted in the composition of the unique image reflecting clostridial population changes. Surprisingly, the main change in expression of individual genes was coupled with the sporulation start and not with the transition from acidogenic to solventogenic metabolism. As expected, solvents formation started at pH decrease and the accumulation of butyric and acetic acids in the cultivation medium.

## Introduction

Strictly anaerobic bacteria represent a less well known and studied group compared to their aerobic or facultatively anaerobic counterparts. Nevertheless, the present need to identify different solutions to problems threatening the ecological and energetic stability of the world has focused attention on these bacteria and has initiated an era of study revealing their powerful metabolic potential. Clostridia, a diverse group of strictly anaerobic bacteria, include known pathogenic and toxinogenic bacteria such as *Clostridium difficile* or *Clostridium botulinum* but also non-pathogenic industrially important species such as *Clostridium acetobutylicum*, *Clostridium beijerinckii* or *Clostridium ljungdahlii*. Among them, butanol-producers, *C*. *acetobutylicum*, *C*. *beijerinckii*, *C*. *saccharoperbutylacetonicum* and others offer a wide range of options related to substrate choice and utilization because they can produce a spectrum of hydrolytic enzymes and can utilize different, often unusual, and insufficiently described metabolic pathways to produce valuable chemical compounds that are currently produced from oil or its derivatives. Current research in the field is focused not only on strain improvement and the use of alternative, waste stream-based substrates but also on acquiring a deeper understanding of clostridial metabolism and life cycle changes. Despite the 100th year anniversary of industrial acetone-butanol-ethanol (ABE) in 2016^1^, butanol producers still managed to surprise us; for recent news in the field, see Herman *et al*. (2017); Jones *et al*. (2018); Sandoval-Espinola *et al*.^2–4^.

The genome of *C*. *beijerinckii* NRRL B-598 was assembled in 2015^5^ having an original species name *Clostridium pasteurianum*. The strain does not contain plasmids, can produce spores, excels in oxygen tolerance and overall fitness, cannot produce isopropanol, and produces very low concentrations of ethanol but high levels of hydrogen during ABE fermentation^6^ Methods for successful transformation of the strain were also described^7^. The strain was proposed for re-identification to *C*. *beijerinckii*^8^ based on this genomic data but also on its similarity with the better known strain *C*. *beijerinckii* NCIMB 8052. Although the strain shares high homologies of most of the key metabolic and life cycle genes with the strain *C*. *beijerinckii* NCIMB 8052, see the comparison of homologies of selected genes^9^, there are undeniable differences in the regulation of gene expressions and probably also at other regulation levels. These differences are best displayed by comparison of transcriptomic data available for both strains (cf. Sedlar *et al*.^10^ and this study for *C*. *beijerinckii* NRRL B-598 with Wang *et al*.^11,12^ for *C*. *beijerinckii* NCIMB 8052). In addition, these differences are manifested in multiple aspects of both populations behaviour such as the proportion of the sporulating population, growth rate, glucose consumption rate or rate of population declination which were mapped for both strains using flow cytometric analysis^13^. For *C*. *beijerinckii* NRRL B-598, it was unambiguously demonstrated^14^, that sporulation is not a necessary condition for solventogenesis and that sporulation can be achieved only under specific culture conditions, defined mainly by the composition of the cultivation medium.

Global population changes observed by transcriptomic profiling during ABE fermentation of *C*. *beijerinckii* NRRL B-598 have already been published^10^. Here, two biological replicates with their respective technical replicates were analysed for changes in expression of individual genes or gene clusters and biological meanings for these expression changes were sought. Mainly, major events during the complete life cycle and both metabolic phases were analysed using different methods and compared with expression changes at the single gene or gene cluster levels to obtain further pieces of the mosaic and thus create a more complete picture of population behaviour in this strain.

## Results and Discussion

**Figure 1:**
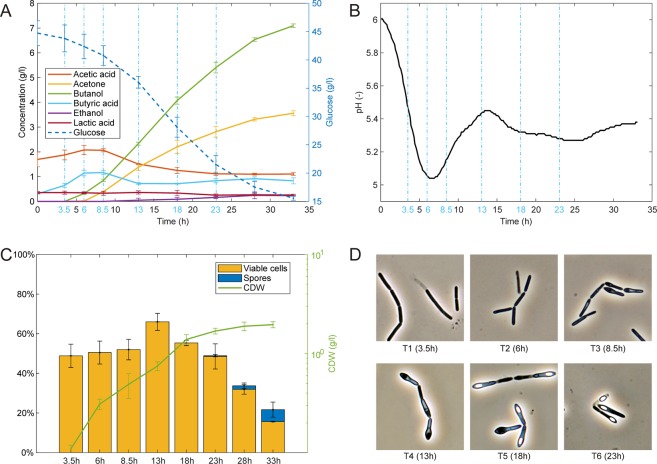
Fermentation characteristics, cell morphology and physiology of *C*. *beijerinckii* NRRL B-598 during ABE fermentation. (**A**) Concentration of glucose and metabolites in medium. (**B**) Course of pH. (**C**) Percentage of active (viable) cells and formed spores based of flow cytometry analysis and growth curve represented by cell dry weight. (**D**) Cell morphology at the moment of sampling for RNA-Seq analysis (magnification 1000x). Error bars represent standard deviations. Sampling points for RNA-Seq analysis are marked with blue vertical dotted lines and/or by blue text labels.

**Figure 2:**
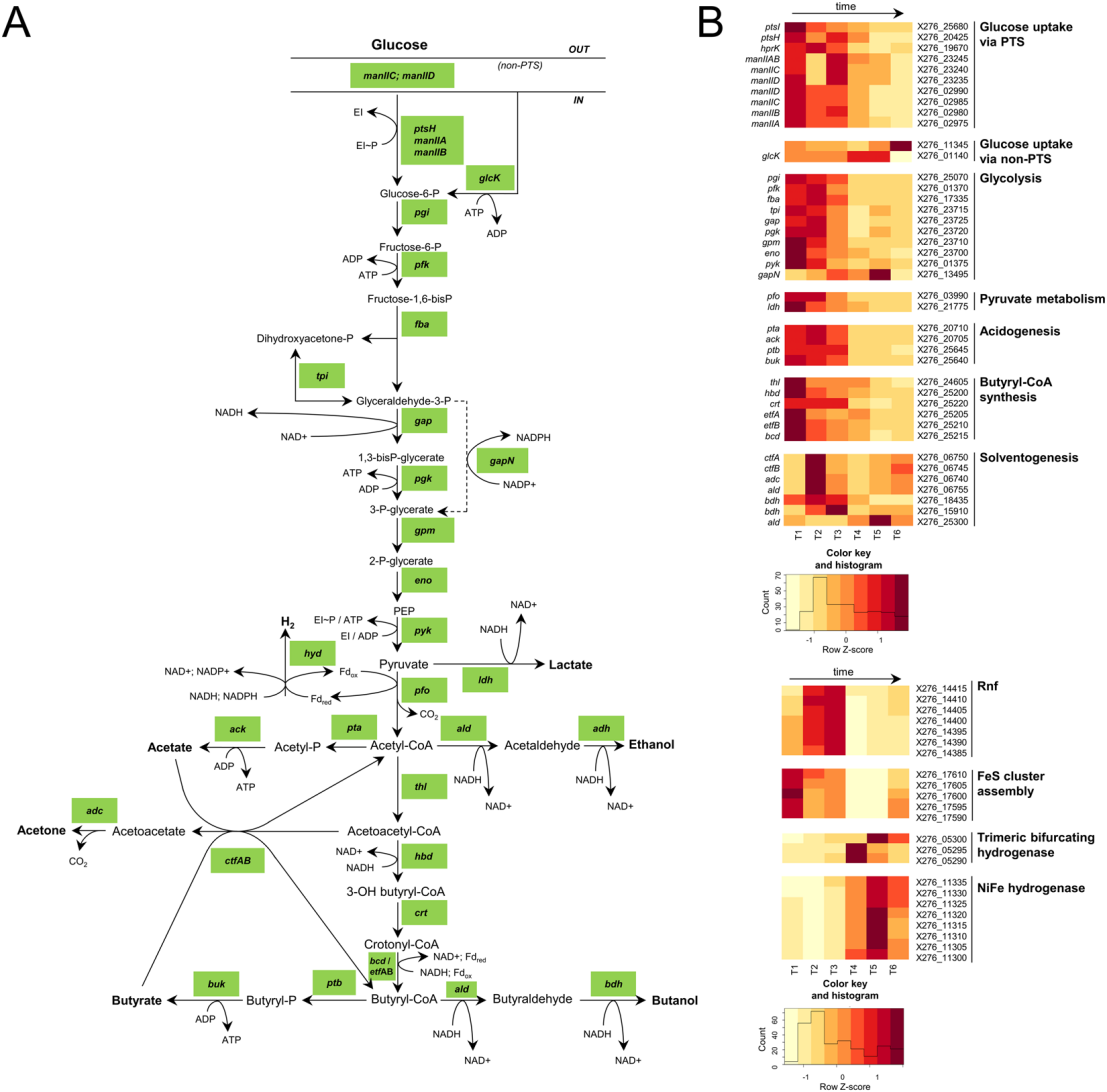
Glucose transport and central catabolism, hydrogen formation. (**A**) Scheme, (**B**) Heatmap displaying changes in transcriptions of the related genes.

**Figure 3:**
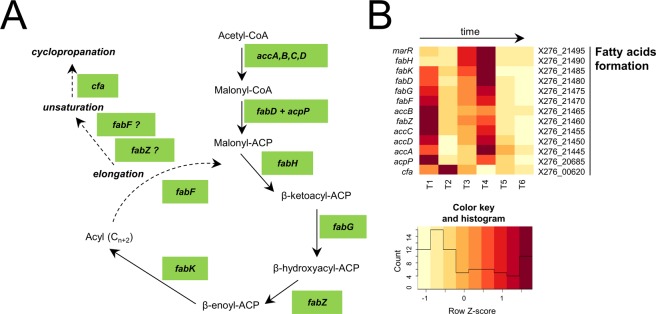
Putative fatty acids formation, (**A**) Scheme, (**B**) Heatmap displaying changes in transcriptions of the related genes.

**Figure 4:**
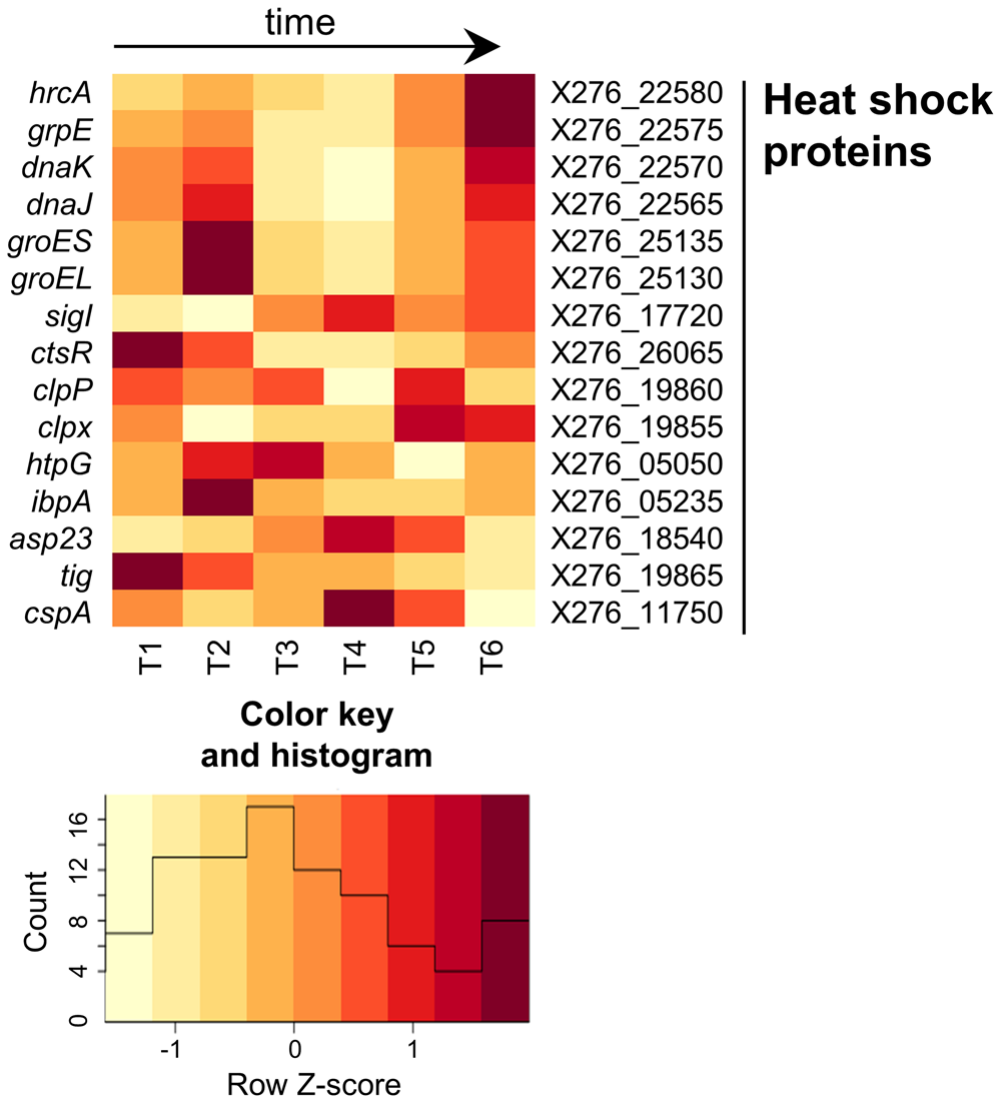
Heatmap of expression changes in genes for heat shock proteins.

**Figure 5:**
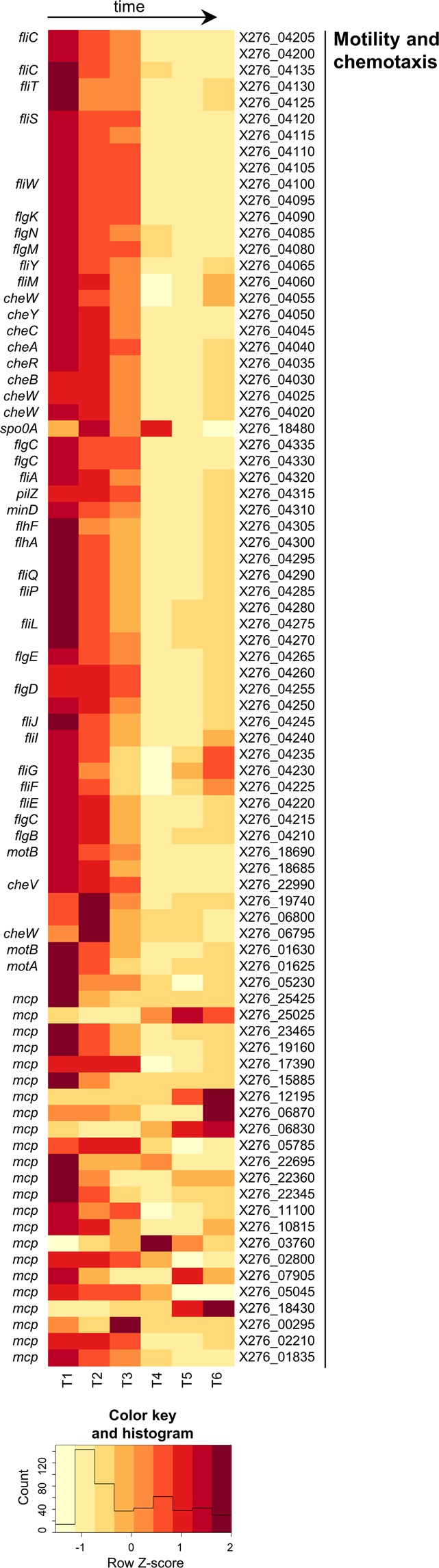
Heatmap of expression changes in genes for motility and chemotaxis.

**Figure 6:**
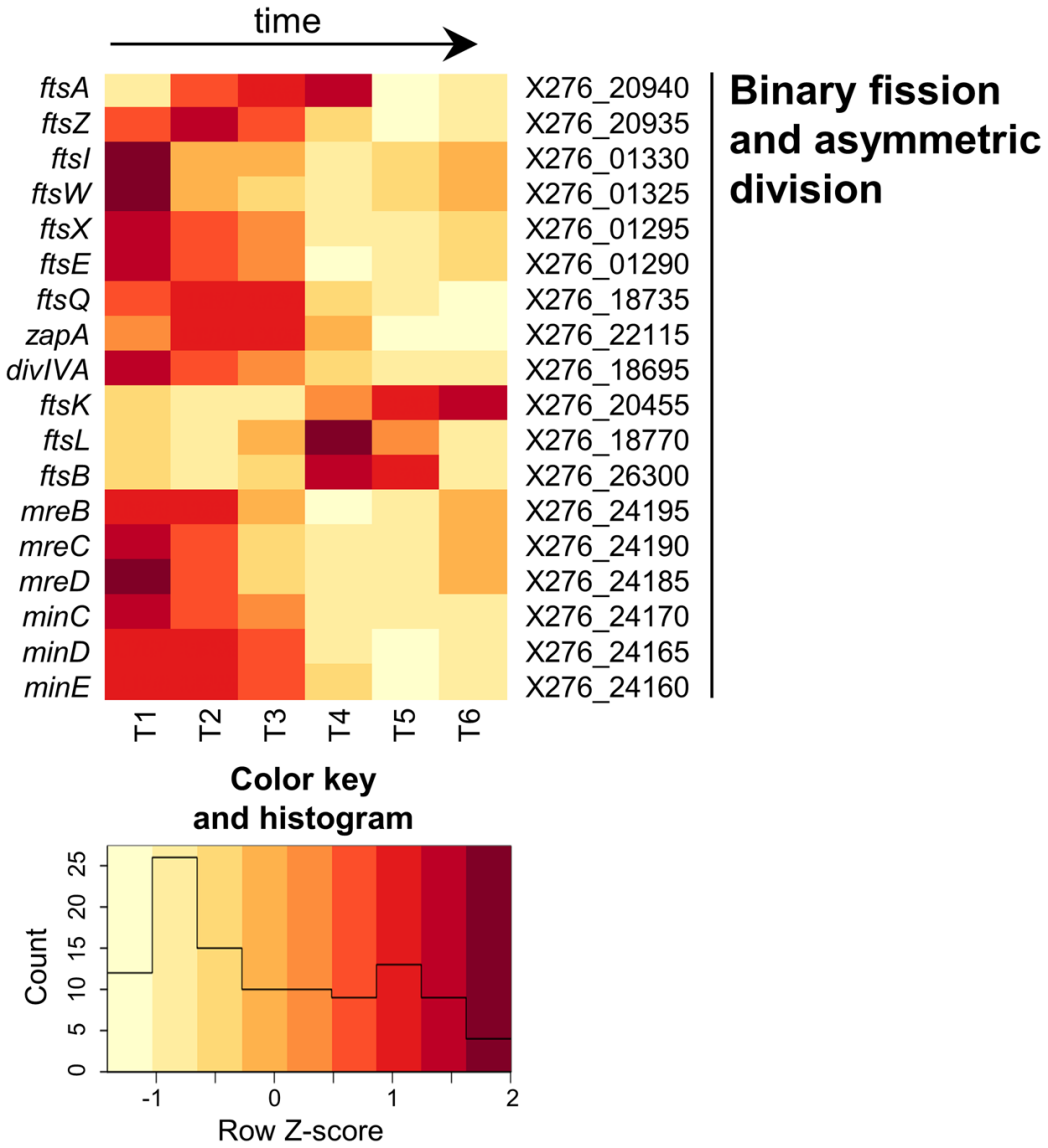
Heatmap of expression changes in genes for binary fission and asymmetric division.

**Figure 7:**
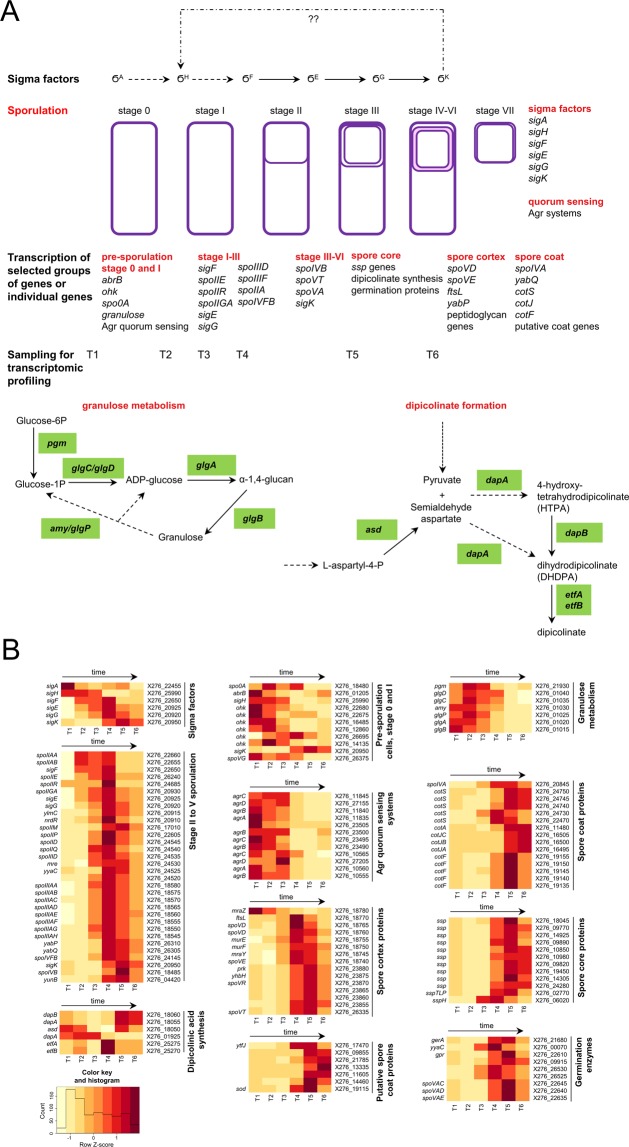
Sporulation, Agr quorum sensing systems, sigma factors, granulose metabolism and dipicolinic acid formation. (**A**) Scheme, (**B**) Heatmap displaying changes in transcriptions of the related genes.

Samples for the *C*. *beijerinckii* NRRL B-598 transcriptomic profiling were taken during batch bioreactor cultivation at time-points T1–T6 to cover the complete cell cycle and both acidogenic and solventogenic phases of ABE fermentation. Data displaying substrate consumption, metabolite formation, pH profile, growth curve, cell morphology and numbers of metabolically active/inactive cells and spores determined by flow cytometry are shown in Fig. 1. Utilization of the same sequencing technology, Illumina NextSeq500, as in the previous case^10^, allowed us to fully verify reproducibility of the experiment design. Although sequencing depth of particular samples ranged from 32.7 to 62.9 million, the majority of sequences were formed by rRNA contamination remaining after lab rRNA depletion. Nevertheless, the number of non-rRNA sequences in particular samples ranged from 5.5 to 18.7, which is still very high coverage (see Suppl_File 1A). A majority of cleansed reads mapped unambiguously to the genome while the number of unmapped reads rose slightly over time. The percentage of multi-mapping reads remained almost the same over time (see Suppl_File 1B). Dimensionality reduction of normalized data using t-Distributed Stochastic Neighbour Embedding (t-SNE) showed that replicates B, C, D, and E were similar to each other at particular sampling times (T1-T6) and demonstrated reproducibility of the data (see Suppl_File 1C). Transcriptional data for individual genes are given in Figs 2–7 in the form of a heatmap using a Z-score related to an average expression of each gene and in Supplementary Files 2–7 as RPKM values (reads per kilobase per million mapped reads) showing RPKM of all four replicates. The supplementary files are numbered in the same way as Figures in the text in order to provide Supplementary Information relating to the same genes expressions of which are shown in the Figures. While a heatmap displays changes in average gene expression among T1-T6 time-points, RPKM values enabled us to compare the coverage of genes between individual samples (B1–E6). Further Supplementary Files 2–7 show results of differential expression analysis (p-adj <0.001) and putative operon organization for particular groups of genes; also putative physiological role or enzyme activity of individual genes are given here. For a majority of genes within the same operon predicted by Genome2D, expression profiles were highly correlated. This confirms correctness of the operon prediction. Yet, in a relatively high number of cases, genes whose transcription was not correlated with remaining genes within a putative operon were also detected. Prediction of affected operons may not be correct or operons may contain another internal promoter. Such a situation was already described for the strain *C*. *beijerinckii* NRRL B-598 in the s*igG* gene that has its own promoter but is also a part of the spoIIG operon^9^. Supplementary Files 2a and 5a display expression of paralogues of some genes involved in central metabolism and motility/chemotaxis, respectively. Gene identification in *C*. *beijerinckii* NRRL B-598 was based on close relationships with *C*. *beijerinckii* NCIMB 8052^8^; further genes were also searched for based on their homologies with those found in *C*. *acetobutylicum* ATCC 824, *Bacillus subtilis* or other model bacteria.

### Carbohydrate transport and catabolism

#### Glucose transport

In *C*. *beijerinckii*, glucose and other hexoses are mainly transported to cells via the phosphoenolpyruvate (PEP)-dependent phosphotransferase system (PTS). This system is multicomponent and consists of two main parts – a histidine-containing protein HPr and enzyme I (EI) – as well as a sugar-specific permease complex (enzyme EII) composed of subunits IIA, IIB, IIC and sometimes IID^15,16^. Simultaneously with transport into the cell, PTS converts carbohydrates into phosphoesters using the phosphoryl group from PEP. Non-PTS glucose transport, mainly using ABC-transporters, is usually of minor significance for *C*. *beijerinckii*^16^.

TYA medium used for cultivation contained glucose as the main carbon source, which is mainly transported to the cells by PTS membrane transport (see Fig. 2A for the scheme). In the *C*. *beijerinckii* NRRL B-598 genome, we identified different PTS systems for transport of different saccharides, which probably share enzyme I protein (X276_25680), histidine-containing protein HPr (X276_20425), and Hpr kinase protein (X276_19670); for their expression profiles see Fig. 2B. These three genes occurred in the genome in single copies, which seems to be common for most Clostridial species^17^. Their essential role in PTS transport was confirmed by their high level of expression over the whole cultivation. Surprisingly, glucose permease complex (enzyme II) genes demonstrated low expression even if the strain was cultivated on glucose. On the other hand, two clusters of genes coding the putative mannose family of enzyme II proteins (X276_23245- X276_23235 and X276_02990- X276_02975) were highly expressed (see Fig. 2B) through the course of cultivation, despite no mannose being present in the medium. This suggested that these mannose PTS systems were the most important for glucose transport in the strain. Also in *C*. *beijerinckii* NCIMB 8052, and its mutant strain BA105, mannose PTS transporters Cbei_0711–0713 and/or Cbei_4557–4560 were highly expressed during cultivation on glucose^12,18,19^ while in the butanol hyperproducing mutant strain BA101, PTS transport was partly impaired, which was compensated for by strengthening of non-PTS glucose transport^19,20^. The importance of the saccharide transport system for metabolite formation was clearly demonstrated for *C*. *beijerinckii* NCIMB 8052 and its mutants. Suppression of mannose PTS used for glucose transport and increased non-PTS glucose transport in BA101 mutant resulted in butanol hyper production^19,21^ while in BA105 mutant^19^, strengthening of mannose PTS used for glucose resulted in massive acids production and eventually an acid crash if the pH was not regulated.

While PTS plays the major role in glucose uptake for *C*. *beijerinckii* NRRL B-598, glucose transport can also occur via non-PTS mechanisms, which are symport and ATP-binding cassette transporters (ABC transporters)^16^. In the genome of *C*. *beijerinckii* NRRL B-598, six genes were annotated as symporters, over 150 genes as ABC transporters, and about 100 genes were described as ABC transporter permeases or transport membrane proteins. Some of these might also be responsible for glucose uptake. For example, the ABC transporter permease (X276_11345) (see Fig. 2B), had high levels of mapped reads and, interestingly, was highly differentially expressed between time-points T4 and T6, while PTS genes that took part in glucose uptake were downregulated between T3 and T5 (see Suppl. File 2). It was assumed for *C*. *beijerinckii* NCIMB 8052 and its mutant BA101 that non-PTS mechanisms played important roles in glucose uptake during the solventogenic phase of growth^21^, and this may also be the case for *C*. *beijerinckii* NRRL B-598. However, further investigation is needed to confirm this assumption. If glucose is transported into the cell via non-PTS routes, glucokinase, GlcK (X276_01140) (see Fig. 2B) will catalyse its phosphorylation to glucose-6-phosphate.

#### Central fermentative metabolism

Most genes involved in central metabolism were differentially expressed during the ABE fermentation (see Suppl. File 2) and their expression, quantified as a Z-score and RPKM, see Fig. 2B and Suppl. File 2, respectively, showed a time dependent behaviour. A putative scheme for glycolysis, acidogenesis, solventogenesis and the formation of hydrogen, including genes that are probably the key ones for these pathways, is displayed in Fig. 2.

Similarly to *C*. *acetobutylicum* ATCC 824 and *C*. *beijerinckii* NCIMB 8052, only one gene for glucose-6-phosphate isomerase (*pgi*) was identified (X276_25070). It showed high expression throughout all analysed periods with the highest expression level being at the first three time-points corresponding to intensively proliferating cells. A similar pattern but with slightly higher regulation at T2 occurred with 6-phosphofructokinase, *pfk* (X276_01370), which is clustered with pyruvate kinase (*pyk*) X276_01375 and is expected to be responsible for fructose-6-P phosphorylation. A considerably high number of mapped reads with comparable regulation was observed for putative ATP-dependent 6-phosphofructokinase (X276_21855). The other gene annotated as *pfk*, X276_23790, had a much lower RPKM value (cf. Suppl. File 2 and Suppl. File 2a-). Two genes for fructose bisphosphate aldolase (*fba*), X276_17335 and X276_11155, were identified. The first one shared 100% identity with Cbei_1903 and 81% with CA_C0827 and is expected to be the major enzyme responsible for formation of trioses from fructose 1,6-bisphosphate^22^. This was confirmed by the number of uniquely mapped reads in RNA-Seq analysis (cf. Suppl. File 2 and Suppl. File 2A). The expression profile of the second *fba*, X276_11155 (see Suppl. File 2a), suggests some contribution of this enzyme in late stages of ABE fermentation.

Genes for the enzymes involved in the subsequent C-3 part of glycolysis (triose-phosphate isomerase - *tpi*, glyceraldehyde-3-phosphate dehydrogenase - *gap*, phosphoglycerate kinase - *pgk* and 2,3-bisphosphoglycerate-independent phosphoglycerate mutase – *gpm*) were clustered together (X276_23725 - X276_23700) in the following order: *gap-pgk-tpi-pgm*- X276_23705-*eno*, where X276_23705 codes for protein kinase (for putative operon organization see Suppl. File 2). According to previous reports^11^, *gap*, *pgi* and *tpi* form an operon with the Cro/Cl family transcriptional regulator (the gene X276_23730 in *C*. *beijerinckii* NRRL B-598). Among the transcripts, *gap* ones were the most abundant, similar to findings for *C*. *acetobutylicum*^23^. The intrinsic terminator downstream of *gap* predicted in *C*. *beijerinckii* NCIMB 8052 or transcript stability^23^ might decrease the number of *pgi* and *tpi* transcripts. Even the *gpm (*X276_23710) and e*no* (X276_23700) genes were probably transcribed independently, which is consistent with the finding of Alsaker and Papoutsakis^24^, they shared similar transcription patterns (according to Z-score change) with higher expression at time-point T1 and a decline at T4–T6. All of these glycolytic genes were highly transcribed at all stages analysed with prevalence in the first part of ABE fermentation. The X276_23705 gene, which is located between *gpm* and *eno*, encodes protein kinase and has an opposite transcription profile with low expression in the exponential growth phase and higher RPKM in stationary growth phase (T4–T5 samples). Phosphoglycerate mutase had three more paralogous genes annotated as *gpm*, but probably these do not participate in glycolysis.

Two genes coding for a *gapN* encoding NADP(^+^) dependent glyceraldehyde-3-phosphate dehydrogenase that catalyses glyceraldehyde-3-P oxidation to 3-P-glycerate without ATP generation were identified. Gene X276_15350 had negligible expression but X276_13495 was highly expressed at the beginning of sporulation, corresponding to a suggested function as a source of NADPH for biosynthetic processes or redox potential modulation^25^. Additionally, NAD(P)H concentration was shown to be important for butanol formation due to the fact that most butanol dehydrogenases are NAD(P)H dependent with low activity in the presence of only NADH^26^.

There are three genes annotated as pyruvate kinase in the *C*. *beijerinckii* NRRL B598 genome, from which *pyk* X276_01375 that is clustered with *pfk* X276_01370 was expected to play the key role in central metabolism. This was verified by its expression level and profile. Putative pyruvate kinase, *pyk* (X276_24225) had a considerably lower number of mapped reads than the major one (cf. Suppl. File 2 and Suppl. File 2a). Nevertheless, the Z-score change indicated in the histogram had the same pattern for both *pyk* (X276_01375 and X276_24225) genes, cf. Fig. 2B and Suppl. File 2a. These genes share 52 and 43% protein sequence similarity, respectively with pyruvate kinase type I (pykF) of *E*. *coli* K-12^27^ which is allosterically regulated by fructose 1,6-bisphosphate. The pykF is supposed to play a more important role in glycolysis than the type II (pykA) that is allosterically activated by AMP, however both types of pyruvate kinases were shown to play a role in control and regulation of glycolysis^28^. Unlike *pyk* X276_01375 and X276_24225, the third DNA sequence annotated as a pyruvate kinase, *pyk* (X276_19650) was expressed predominantly at T4 and T5, corresponding to stationary phase and sporulation (see Suppl. File 2a). The expression profiles of such three *pyk* paralogues were described recently by Wang *et al*. (2012) for *C*. *beijerinckii* NCIMB 8052^12^.

Generally, expression of all predicted major enzymes involved in transformation of fructose-6-P to pyruvate through glycolysis was high and declined over the course of fermentation, which is in agreement with previous findings for *C*. *beijerinckii* NCIMB 8052 and *C*. *beijerinckii* BA101^11,19^ but in contrast with the transcriptomic analysis of *C*. *acetobutylicum* ATCC 824 where these genes were not temporally regulated^29^ or were even more highly expressed in stationary growth phase^24^.

Formation of acetyl-CoA from pyruvate in *C*. *beijerinckii* strains is catalysed by pyruvate:ferredoxin (flavodoxin) oxidoreductase (*pfo*)^30^. The *pfo* X276_03990 shared 99% identity with Cbei_4318 and 72% with CA_C2229, likely dominant *pfo* genes in related strains *C*. *beijerinckii* NCIMB 8052 and *C*. *acetobutylicum* ATCC 824^26^. Another two homologous *pfo* (see Suppl. File 2a) had substantially lower RPKM values and were transcribed preferentially in stationary phase similarly to transcriptomic profiling of *C*. *beijerinckii* DSM6423^31^.

Regardless of whether it was acidogenic or early solventogenic stage, all of the key genes in acid and solvent formation pathways were more highly transcribed within the first 8 hours of cultivation, similar to glycolytic genes. Moreover, it should be noted that the RPKM values of most of them were significantly higher than their homologous genes that were annotated to have the same function but were regulated at different time-points (cf. Suppl. File 2 and Suppl. File 2a).

Genes for acetic and butyric acid formation from their respective CoA precursors were reported to be organized in a bi-cistronic operon structure^30^, and the same applied for *C*. *beijerinckii* NRRL B-598 (see Suppl. File 2, operon_0636 and operon_0105, resp.). Phosphate acetyltransferase (*pta)* (X276_20710) and acetate kinase (*ack)* (X276_20705) genes coding for acetate formation from acetyl-CoA were only found in one copy in the genome. The whole operon (no. 0636 in Suppl. File 2) was more highly transcribed at the first three time-points T1–T3 with the maximum transcripts at T2 corresponding to the acidogenesis/solventogenesis transition. A similar expression pattern was observed for *ptb* (X276_25645) - *buk* (X276_25640) clustered genes (operon_0105), whereas according to Z-score change *ptb* had equal expression at T1–T3 time-points and *buk* showed a decrease in expression since the first measured point. Unlike *C*. *beijerinckii* strain DSM6423^31^, *pta-ack* and *ptb-buk* in *C*. *beijerinckii* NRRL B-598 were differentially expressed (Suppl. File 2); *pta-ack* between all T2–T4 and T5–T6 adjacent points and *ptb-buk* were surprisingly not regulated identically. Jones *et al*. (2008) reported that in *C*. *acetobutylicum* 824 *pta-ack* were not regulated during ABE fermentation and *ptb-buk* were upregulated at late exponential phase^29^. The latter was also described by Alsaker and Papoutsakis (2005), who measured elevated transcription of *pta-ack* during metabolic transition^24^. In addition, there were two more homologous *buk* genes annotated as butyrate kinase in the *C*. *beijerinckii* NRRL B-598 genome, namely X276_05815, and X276_02775, which had very low transcriptional levels compared to X276_25640, see Suppl. File 2a.

The first step in butyryl-CoA synthesis from acetyl-CoA consists of condensation of two acetyl-CoA molecules and is catalysed by acetyl-coenzyme A acetyltransferases (thiolase, *thl*). Two acetyl-coenzyme A acetyltransferases were annotated in the genome but only one of them (X276_24605) was transcribed, consistent with previous findings^12^. The Thl is the only enzyme from this pathway whose gene was not expected to be organised in the *bcs* operon. Members of the putative *bcs* (butyryl-CoA synthesis) operon were clustered together in the following order: crotonase (*crt*)- butyryl-CoA dehydrogenase (*bcd*) – *etfB* – *etfA* - 3-hydroxybutyryl-CoA dehydrogenase (*hbd*), (X276_25220 - X276_25200). Surprisingly, Genome2D operon prediction suggested another operon organisation and placed the last gene *hbd* (X276_25200) into a separate operon (operon_0155). Simultaneously, expression profiles showed high correlation of the *hbd* with genes X276_25215–35 but not with X276_25220 (*crt*), see Suppl. File 2. The *bcs* operon (in *C*. *beijerinckii* NRRL B-598 operon_0154) includes genes for the electron-transferring flavoprotein complex EtfAB, which, in the presence of NADH, catalyzes crotonyl-CoA dependent reduction of ferredoxin and simultaneous formation of butyryl-CoA^32^. The expression of these genes was high and corresponded to the expression profile of thiolase. All of them (except *crt*) were mostly expressed at time-point T1, corresponding to intensive growth and acid formation, however RPKM values remained high until the last time-point measured. Similar gene clusters with locus tags X276_16680 - X276_16660 were found within the genome. While four genes shared more than 99% sequence similarity, the last genes of these operons, *hbd*, X276_25200 and X276_16660, were unique. Because the number of reads mapping to X276_16660 was very low, this operon was probably silent and expression of preceding genes was false positive caused by multi-mapping reads from the active *bcs* operon.

Re-assimilation of acids is catalysed by CoA-transferase, consisting of two subunits, A (*ctfA*) and B (*ctfB*), which transfer CoA from acetoacetyl-CoA to acetate or butyrate. Three such transferases were identified in *C*. *beijerinckii* NRRL B-598 but only one, X276_06750/ X276_06745, was located in the *sol* operon (*ald-ctfA-ctfB-adc*) adjacent to acetoacetate decarboxylase (*adc*) X276_06740 and aldehyde dehydrogenase (*ald*) X276_06755 genes, see Suppl. File 2, operon 2326. The expression profile (see Fig. 2B) revealed the expected transcriptional switch of the solventogenesis-related genes at the pH breakpoint^19^ with coordinated regulation.

Ethanol and butanol are formed from acetyl-CoA or butyryl-CoA respectively via dehydrogenation reactions that in *C*. *acetobutylicum* ATCC 824 are catalysed by bifunctional alcohol/aldehyde dehydrogenases AdhE1 and AdhE2, and in *C*. *beijerinckii* NCIMB 8052, by two separate enzymatic activities of Ald (aldehyde dehydrogenase) and Bdh (Adh) (butanol (alcohol) dehydrogenase). *C*. *beijerinckii* strain NRRL B-598 formed a very small amount of ethanol during fermentation, indicating that the pathway of ethanol formation was retarded. Butanol was produced at low titres even before the onset of solventogenesis and was intensified after the metabolic switch. Among a wide range of alcohol and aldehyde dehydrogenases present in the *C*. *beijerinckii* genome^12^ one was annotated as a bi-functional acetaldehyde-CoA/alcohol dehydrogenase (X276_25300) and is probably the counterpart of AdhE2. It was upregulated in later stages (T4, T5) but had a considerably lower number of transcripts detected by RNA-Seq. Aldehyde dehydrogenase, *ald* gene (X276_06755) corresponding to *adhE1* according to its localization in the *sol* operon had high numbers of uniquely mapped reads and was co-expressed with *ctf*A/B and respective solventogenic genes (see Fig. 2B and Suppl. File 2). This therefore is apparently the key enzyme in the formation of butyraldehyde from butyryl-CoA. The subsequent step in butyraldehyde reduction to butanol might be performed by various different dehydrogenases. In a related strain, *C*. *beijerinckii* NCIMB 8052, the enzyme encoded by gene Cbei_2421 should take over the function of BdhI,II described in *C*. *acetobutylicum* based on a similarity analysis of the product^22^. A homologous gene (X276_14550) with 99% homology to Cbei_2421 was actively transcribed during fermentation but RPKM values suggested that this was not a butanol dehydrogenase playing the key role in butanol formation (see Suppl. File 2a). Within the genes annotated as alcohol dehydrogenases, some of them were more specifically annotated (in the ISNDC database) as putative butanol dehydrogenases and from these, X276_18435 had the highest level of expression. A reasonable level of transcripts was also associated with *bdh* X276_15910 that was upregulated at the metabolic switch. These results are consistent with the findings of Wang *et al*. (2012), who identified the two most transcribed iron-containing dehydrogenases that were homologous with identified *bdh*, Cbei_1722 and Cbei_2181 respectively^12^.

Wang *et al*. (2012) described the abundance of isoenzymes in *C*. *beijerinckii* NCIMB 8052 sharing the same function but with altered expression patterns at different stages^12^. For the central metabolic genes analysed in our study, it was shown that even when these homologous enzymes with the same putative functions were expressed at different periods, the expression level of “key” enzymes was much higher, suggesting that these isoenzymes play only minor roles in metabolic changes under standard ABE fermentation. While the expression of key enzymes is shown in Fig. 2B, expression of putative paralogues of some of these is displayed in Suppl. File 2a.

#### Hydrogen formation

In *C*. *beijerinckii*, reduced ferredoxin might be regenerated in two possible ways, by formation of H_2_ through the action of hydrogenases, or by translocation of protons through the Rnf complex. Genes coding for the Rnf complex were clustered together (X276_14415 – 75) in an operon structure (see Suppl. File 2), and were highly expressed at T2 and T3, namely at the pH breakpoint and early solventogenesis (see Fig. 2B). This suggests the main contribution of the Rnf complex was in solventogenesis. On the other hand, production of H_2_ by hydrogenases was unclear. For *C*. *beijerinckii* strains, a trimeric bifurcating hydrogenase has been described^30^ that might generate hydrogen from electrons from NADH and ferredoxin simultaneously^22^. Such a trimeric bifurcating hydrogenase was found in *C*. *beijerinckii* NCIMB 8052, encoded by genes Cbei_4410 – Cbei_4412. These shared high similarity with *C*. *beijerinckii* NRRL B-598 strain orthologues (X276_05300 – 35). A similar organisation of this bifurcating hydrogenase was found in more Clostridial strains, e.g. *C*. *butyricum*^33^ but according to our transcriptomic data, this gene cluster was not transcribed sufficiently to possess sufficient electron flux (see Suppl. File 2). While *pfo* had RPKM values in the range of thousands, the bifurcating hydrogenase was mostly less than ten RPKM (see Suppl. File 2). Actually, none of the genes annotated as hydrogenase had presumptive expression (see Suppl. File 2b). In accordance with findings of Calusinska *et al*.^34^, diverse hydrogenases were found in the *C*. *beijerinckii* NRRL B-598 genome. Components of one [NiFe] hydrogenase complex were identified and were clustered together (X276_11335-720); the putative large subunit (X276_11305) also contained characteristics motifs RICGICSTAH and DPCxSCATH conserved in clostridial [NiFe] hydrogenases. Moreover, Hyp regulation proteins were also present in the cluster. In clostridia, Hyp proteins regulate expression of structural genes^34^. However, the [NiFe] hydrogenase gene cluster revealed the highest expression in stationary growth phase (T4, T5) and low transcription in the acidogenic stage when intensive production of H_2_ occurred (see Fig. 2B). To reveal which hydrogenase contributed to hydrogen formation in *C*. *beijerinckii* NRRL B-598, homologous genes were searched according to similarity with hydrogenases described in *C*. *beijerinckii* strain NCIMB 8052. Genes with locus tags X276_17350, X276_06930 and X276_05300 shared high similarity with Cbei_1901, Cbei_3796 and Cbei_4110 respectively; none of these genes had a reasonable RPKM value (RPKM <100) except the putative hydrogenase gene X276_17350 during stationary phase, where it was upregulated (see Suppl. File 2a). Among hydrogenase maturation and regulation proteins with detectable expression, X276_09925, X276_03475 and X276_03470 had higher expression at the solventogenesis switch point and X276_25305 at time-point T4 (see Suppl. File 2a). According to transcriptomic data, we hypothesise that during exponential growth phase, among putative ferredoxins, ferredoxin with locus tag X276_26075 plays the major role in electron flux from central metabolism. The analogous expression profile to this ferredoxin was identified for a cluster of genes for the assembly machinery of FeS domain proteins (X276_17610 - X276_17590). It was also clustered with *pfo* X276_17585 but this *pfo* isoenzyme had a low expression level and was 396 bp distant from the complex. Unfortunately, based on available transcriptomic data, we have not managed to unambiguously detect which gene was responsible for production of a functional hydrogenase that produces H_2_ in *C*. *beijerinckii* NRRL B-598 in exponential growth phase. A potential candidate might be hydrogenase X276_18165 (see Suppl. File 2a), which shares 99% homology with that isolated from *C*. *beijerinckii* SM10^35^. This hydrogenase is one of the group of enzymes annotated as 4Fe-4S dicluster domain containing proteins (for expression see Suppl. File 2a). but is the only one with a matching expression profile.

### Stress responses induced by solvent and/or acid formation

#### Changes in fatty acids metabolism

Changes in fatty acid saturation and length represent one of the first explored mechanisms of overcoming heat and solvent stress in bacterial cells. Higher saturation, as well as extension of length of fatty acids bound in membrane lipids, can stabilize bacterial membranes, and increases in the concentration of these fatty acids could generally be detected after the start of butanol biosynthesis in clostridia^36–38^. The transition of *cis* to *trans* conformations of double bonds is another known reaction to heat and solvent shock in bacteria; while *cis* conformation typically gives bent molecules, the *trans* conformation is more similar to the spatial arrangement of saturated hydrocarbon chains and promotes membrane stabilization^39,40^. Clostridia produce the same species of unsaturated fatty acids as the model Gram-negative bacterium *E*. *coli*, however they lack the known enzymes for their biosynthesis. Anaerobes also cannot use molecular oxygen as do aerobic bacteria during unsaturation reactions^41,42^. The main genomic region that is responsible for fatty acid biosynthesis is the *fab* operon, where all necessary genes for fatty acid biosynthesis are located. The *fab* operon of *C*. *acetobutylicum* lack genes *fabM*, *fabA* and *fabB* compared to *E*. *coli* and three putative *fabF* genes could be found across the genome while only one is probably involved in fatty acid production and is located in the *fab* operon. The *fabF* is also the gene whose product is able to replace the function of FabB and is most probably responsible for unsaturated fatty acid biosynthesis in clostridia^43^.

In *C*. *beijerinckii* NRRL B-598, the fatty acid biosynthetic cluster of genes is the most probably organized in four operons (0525, 0526, 0527 and 0528) as predicted by the *in silico* operon prediction tool (see Suppl. File 3). This organization seems to be typical for *C*. *beijerinckii* species as well as e.g. *B*. *subtilis* model^44^. On the other hand, it is in contrast with *C*. *acetobutylicum* ATCC 824 that have all genes organized in one single *fab* operon^45^. Similarly, unlike in *C*. *acetobutylicum*, there was no gene coding acyl-carrier protein (ACP) in the mentioned fatty acid biosynthetic genes region but the gene *acpP* (X276_20685) was located out of the cluster individually as well as in *C*. *beijerinckii* NCIMB 8052^11,45^. The postulated biosynthetic pathway for fatty acid biosynthesis in *C*. *beijerinckii* NRRL B-598 is presented in Fig. 3A, and changes in expression of related genes are shown in Fig. 3B.

*Clostridium* species normally lack the gene coding the *cis*/*trans* isomerase, so this mechanism is not involved in butanol resistance in *C*. *beijerinckii* NRRL B-598. An additional type of cellular reaction to solvents is the production of cyclopropane fatty acids, as was shown in *C*. *acetobutylicum* ATCC 824^38^. Cyclopropane fatty acids are generally present in all Clostridial spp. and represent a relatively large fraction of standard membrane bound fatty acids. In *C*. *acetobutylicum*, cyclopropane fatty acids replace unsaturated fatty acids when butanol is present, which probably leads to higher resistance and better maintenance of membrane functions during solvent stress^37,38^. Based on the previously published dynamic simulation, cyclopropane fatty acids probably make membranes more stable because of a limitation in rotation around the cyclopropane moieties, but simultaneously they preserve or even increase fluidity, which is necessary for correct membrane function^46^. Cyclopropane fatty acids are synthesized through methylenation of the double bond of particular unsaturated fatty acids by the action of the enzyme cyclopropane-fatty-acid-synthetase, encoded by the *cfa* gene^47^. A deficiency in the *cfa* gene can induce decreased resistance to solvents, and potentially also acids, as was shown in *E*. *coli* or *Pseudomonas putida* mutant strains^48,49^. The *C*. *acetobutylicum* mutant strain with over-expressed *cfa* showed significantly higher resistance to butanol, more effective growth in the presence of butanol and higher resistance to butyric acid. Unfortunately, butanol production was disturbed at the same time^38^.

It is clear that the highest level of expression of the genes responsible for fatty acids biosynthesis in *C*. *beijerinckii* NRRL B-598 was established at the beginning of cultivation (T1) and from T3 to T4, see Fig. 3B. This seems to reflect both acids formation (T1) and solvents production (T3, T4), cf. Fig. 1A,B i.e. metabolites formation and pH profile. On the other hand, at sampling time-points T2, T5 and T6, expression was, conversely, very low. This fits very well with the expression results of the *acpP* gene (X276_20685), whose expression maxima matched the maxima of fatty acid biosynthetic cluster genes, see Fig. 3B. Expression of the *cfa* (X276_00620) gene was the highest at times T1–T3 with a local maximum at time T2. This was also consistent with the fact that Cfa can transform matured unsaturated fatty acids whose biosynthetic genes are expressed with a local maximum at T1. Furthermore, T2 represents the first time-point with clearly detected butanol production, which could represent an important trigger for changes in fatty acid metabolism genes expression, even at low concentration suggesting that *cfa* expression could be regulated by the presence of butanol or potentially the presence of unsaturated fatty acids. After point T2 there was visible perceptible reduction in *cfa* expression. Phospholipid-bound fatty acid composition during standard ABE cultivation was also analysed previously in *C*. *beijerinckii* NRRL B-598, using orbitrap mass spectrometry^36^. This analysis revealed the highest percentage of cyclopropane dimethylacetals, which were derived from fatty acids bound in plasmalogen phospholipids (containing a vinyl-ether linkage at the sn-1 position and ester linked sn-2 position), at 6 hours after inoculation, which corresponded with T2 in this work. The percentages of cyclopropane fatty acids derived from diacylglycerol phospholipids were relatively stable. This shows that cyclopropane fatty acids synthesized by Cfa are probably preferentially used for plasmalogen lipid formation in *C*. *beijerinckii* NRRL B-598, which is probably a common trend for bacteria, especially under stress conditions^50^.

#### Heat shock protein metabolism

Heat shock proteins (HSPs) represent a family of proteins that are transcribed when heat or other general stresses, including accumulation of metabolites, acids or solvents, are detected by the bacterial cell response mechanism. HSPs mostly function as molecular chaperons that are able to help other proteins fold into their native conformations, but among HSPs we can also find proteases, proteins stabilizing DNA conformation and proteins having many other effects^51^. Four classes of HSPs have been described in Gram positive bacteria to date^52^. Class I HSPs are controlled by negative regulation, mediated by HrcA regulators that are bound to CIRCE elements on DNA sequences. HrcA probably needs to interact with GroES/EL chaperonins for active binding to CIRCE^53,54^). All molecules of these chaperonins are rapidly bound to unfolded proteins in the cytosol when heat shock is established, which leads to HrcA inactivation and initiates expression of the next class I HSPs^53^ (Mogk *et al*., 1997). Two important operons are known to belong to class I HSPs: DnaKJ and GroESL, and these are found in solventogenic clostridia, including *C*. *beijerinckii*.

Class II HSPs are characterized by expression that is controlled by the alternative sigma factor SigB in *E*. *coli* and *B*. *subtilis*. A *sigB* homologue has not been found in clostridia and potential class II HSP orthologues differ significantly, are missing or are not transcribed^55^, which led to the theory that class II HSPs do not play an important role in solventogenesis or heat shock. Alternative sigma factor SigI, which has been found in many clostridial genomes including *C*. *acetobutylicum* (CA_C1770), could partially substitute for this function in clostridial regulation of HPSs. SigI probably co-regulates expression of several HSPs and other proteins and its mutation leads to unambiguously lower heat resistance in *B*. *subtilis*^56^ or to disruption of toxin production in *Bacillus anthracis*^57^.

Class III HSPs represent the next family of small HSPs with negative regulation of expression but these are not regulated by HrcA nor CIRCE. One of the most common negative expression regulators is probably CtsR, which is able to regulate expression of several stress proteins when heat or oxidative shock occurs^58,59^. Known examples of CtsR regulated genes are *clpX*, *clpP* and genes of the *clpC* operon in *B*. *subtilis*. In *C*. *acetobutylicum*, the same consensus sequence for CtsR binding has been found for the *clpC* operon and Hsp18 but not for heat inducible chaperones/proteases ClpX and ClpP^45^ whose expression is probably regulated in different and still unknown ways. HSPs belonging to class IV are regulated by at least two regulators and there is little information about them. For example, HptG, YdkA, Asp23 and other proteins are probably included in this group.

Operons of class I HSPs, DnaKJ and GroESL, are relatively conserved in prokaryotic organisms and the same operon architecture can be found in the model bacteria *E*. *coli* and *B*. *subtilis* as well as in clostridia. Classic organization of GroESL operon (operon 0165) has also been found in *C*. *beijerinckii* NRRL B-598: *groES* (X276_25135)*-groEL* (X276_25130), see Suppl. File 4. Genes *hrcA* (X276_22580)*-grpE* (X276_22575)*-dnaK* (X276_22570)-*dnaJ* (X276_22565) are also located in the same genomic region in the *C*. *beijerinckii* NRRL B-598 however a relatively long, non-coding DNA fragment is inserted between *dnaK* and *dnaJ* genes. Because of this fact, both genes were designed in various operons by *in silico* analysis (operons 0444 and 0445, see Suppl. File 4), but it is possible that they form only one operon because no satisfactory promoter prediction was found prior to the *dnaJ* gene; RNA-Seq data also correlate with this assumption. As described above, the *hrcA* gene product is responsible for regulating expression of the class I HSPs operons, and DnaK (also known as Hsp70) and DnaJ (Hsp40) are classic molecular chaperons that mainly protect unfolded proteins prior to irreversible aggregation. The GroEL/ES complex of chaperonins plays a significant role especially in the refolding of misfolded proteins.

Based on the RNA-Seq data, it is evident that the strongest expression of class I HSPs occurred at two local maxima: time-points T2 and T6 (see Fig. 4). This suggests that the highest concentration of unfolded proteins in the cytosol occurred at these time-points because all molecules of GroES/GroEL are bound to unfolded proteins that can unblock HrcA-mediated negative regulation and activate gene expression. Time-point T2 was characterized by the first detected butanol production as well as by a minimum pH of the medium and in T6 by a high concentration of solvents and a second pH minimum. This could indicate that production of class I HSPs is connected especially to pH stress induced by the production of organic acids, see Fig. 1A,B. Expression of SigI seems to be higher with increasing titer of butanol in the medium as well as prolonged time of cultivation.

The homologue to alternative sigma factor SigI, which might be related to expression of HSP class II, was found in *C*. *beijerinckii* NRRL B-598 (X276_17720). Its expression increased with time of cultivation, with local maxima at times T4 and T6 (see Fig. 4), which could indicate some function in overcoming solvent stress. On the other hand, no convincing orthologues of *Bacillus* class II HSPs were found in the genome of *C*. *beijerinckii* NRRL B-598 based on the homology analyses and some of the orthologues found in *C*. *acetobutylicum* ATCC 824^55^ were missing completely e.g. CA_C1514 or CA_C1414.

The main, negative regulator of class III HSPs, CtsR (X276_26065), was expressed strongly at the beginning of cultivation and its expression decreased with time and with higher butanol titer in culture (see Fig. 4). This could indicate that class III HSPs expression is activated when butanol is produced and that these proteins can play a role in overcoming butanol stress in *C*. *beijerinckii* NRRL B-598. The uncategorized HSP, the gene *hptG* (X276_05050), which has been previously shown to be one of the main candidate genes for overcoming butanol stress in clostridia^60^, was expressed strongly when butanol production began, so it is probable that it was also involved in overcoming butanol shock in *C*. *beijerinckii* NRRL B-598. A similar expression profile was also observed for some other, uncategorized potential HSPs such as X276_05235, X276_05235, X276_19865 and others (see Fig. 4).

It has been proven previously that over-expression of genes encoding HSPs can lead to significant increase in butanol tolerance in *E*. *coli*^61^ as well as to increase in butanol tolerance and/or its production in clostridium bacteria^62^. Tomas *et al*. (2004) reported that the highest expression of several HSPs, including GroES/EL and DnaK/J, fits positively with solvent production in *C*. *acetobutylicum* ATCC 824^45^. Furthermore, overexpression of GroES/EL resulted in a significant improvement in butanol tolerance and higher butanol production in the same bacteria^63^. Mutant strains with overexpression of GrpE or HptG HSPs were also prepared, which led to prolonged survival of bacterial cells in the presence of 2% butanol^60^.

### Life cycle changes

For the purpose of this manuscript, life cycle changes that are typical for sporulating solventogenic clostridia are divided into three groups: motility/chemotaxis, binary fission and sporulation. A typical life cycle of *C*. *beijerinckii* NRRL B-598 exhibited the following phases, most of which could be observed under the microscope (see Fig. 1D):

1. after spore germination, the cells grew in long chains of rod cells (both spore germination and chain formation were performed during growth of the inoculum and these changes were not reflected during RNA-Seq analysis),
2. the chains broke down into individual cells, part of them being highly motile and performing “runs” during movement (time-point T1)
3. there was a phase of culture growth specified by binary fission of vegetative cells; some of the cells were motile (time-point T2)
4. granulose accumulation, which gives cells their typical “clostridium-like” swollen shape, but there were still some motile cells (time-point T3)
5. initiation of formation of spore septum; if there are motile cells in the sample, they “tumble” (time-point T4)
6. visible bright pre-spores in mother cells (time-point T5)
7. visible bright spores in mother cells, sporadic release of mature spores from mother cells (time-point T6)

However, it must be mentioned that individual cells in the population during batch cultivation did not proceed simultaneously and there was always some vegetative, even motile cells throughout the whole cultivation; also sporulation did not proceed in a synchronous way. Population analysis performed by flow cytometry and staining is shown in Fig. 1C. The main life cycle changes, sorted into motility/chemotaxis, binary fission/asymmetric division and sporulation, are described at the transcriptomic level in the following sections. As many genes (e.g. those coding the Fts family of proteins) involved in septum formation during binary fission also take part in formation of the spore septum, asymmetric division associated with septum formation is discussed together with binary fission in section 2.3.2. while other sporulation gene transcriptions are described separately in section 2.3.3.

#### Motility and chemotaxis

Motility is a substantial and pronounced feature that can be observed in young *C*. *beijerinckii* cultures shortly after germination of spores. Motile cells form peritrichal long flagella that enable typical cell swirling. From the very beginning of ABE fermentation research and industrial practice, motility was associated with viability, especially in case of inoculum, fitness and a good chance to obtain a high solvent yield. The main reason for bacterial motility is probably chemotaxis, which can be either positive (toward an attractant such as sugar) or negative (from a repellent such as acid, solvent or oxygen). In *C*. *beijerinckii* P262^64^, during positive chemotaxis, typical “runs”, i.e. counterclockwise smooth movement, were observed, while during negative chemotaxis, “tumbling” i.e. clockwise rotary movement, prevailed.

The phenomenon of chemotaxis is impossible to study in a homogenous bioreactor environment during mixing, but motility was observed in part of the population under the microscope, mostly from time-points T1 to T3. As flagella building is an energy demanding process, which actually does not bring any advantage over sessile cells in a bioreactor environment, it seems that motility of part of the population is a part of a bet-hedging strategy. If living conditions go bad, part of the population will be prepared to escape actively. A similar strategy was described for *B*. *subtilis*^65^, however not all motile bacteria behave the same because it is also smart to give up motility when living conditions are good.

A bacterial flagellum consists of a motor and a stator forming together a flagellar basal body plus flagellar hook functioning as a joint and a filament^66^. All main genes involved in flagellum assembly were identified as follows: two homologues of genes for stator formation *motB* (X276_01630; X276_18690), *motA* (X276_01625, X276_18685) in operons 0857 and 2892; rotor proteins genes *fliF* (X276_04225), *fliG* (X276_04230), *fliM* (X276_04060) with the exception of *fliN*; hook formation genes *fliE* (X276_04220), *flgE* (X276_04265); flagellin genes *fliC* (X276_04205, X276_04135) and their regulatory proteins - specific alternative sigma factor FliA (X276_04320) and anti-sigma flagellar factor FlgM (X276_04080). Most flagellum related genes (see Fig. 5) were transcribed especially in time period T1–T3 and were downregulated from time-point T2; for their interesting putative operon organization and differential expression analysis see Suppl. File 5. It is also noteworthy that the flagellum related genes were mostly upregulated at time-point T6 (see Suppl. File 5) which might be caused by the worsening living conditions in a bioreactor and an effort of some cells in the population to escape. It was described for *B*. *subtilis*^67^ that early flagellar genes involved in formation of the flagellar basal body and hook formation are transcribed under control of SigA factor while late flagellar genes (mainly flagellin formation) are under the control of FliA factor, which is released from its bonding with FlgM anti-factor. However, expression profiles of flagellar genes identified in our strain did not match these findings, see Fig. 5. It seems more probable that FlgM might be involved in control of flagella number per cell, as in *Rhodobacter sphaeroides*^68^.

In motile cells, movement in a specific direction is initiated by accepting a signal from the environment, which results in methylation of methyl accepting chemotaxis protein (Mcp). Many membrane receptors (*mcp* genes) were identified in the genome and their individual transcription peaks were noted over the whole cultivation (from T1 to T6) (see Fig. 5). Methylated Mcp transfers the signal to Che protein phosphorelay cascade, which results in phosphorylation of the motility main regulator CheY. In some bacteria (Szurmant and Ordal, 2004), another regulator, Che-Y^*^ is formed, which interacts with Che-Y-P and functions as a phosphate sink. Actually, it cannot be excluded that Spo0A global regulator (X276_18480) plays several different roles in the clostridial life cycle and functions as both a negative motility/chemotaxis regulator (see upregulation of its expression between time-point T2 and T1 in Suppl. File 5) and positive regulator of sporulation. The *fla/che* operon of *B*. *subtilis*^69^ was demonstrated to be negatively regulated by Spo0A, however in *C*. *beijerinckii* NRRL B-598, the operon organization of orthologous genes was different. A negative influence of *spo0A* overexpression was observed for *C*. *acetobutylicum*^70^. The CheY-P protein interacted with a component of the flagellar motor and switched the movement. All main parts of the Che phosphorelay system depicted by Szurmant and Ordal^71^ were found in two adjacent operons (operons 2614 and 2615; adjacent genes from X276_04055 to X276_04020) with the exceptions of *cheW* orthologue (X276_06795) and *cheV* gene (X276_22990). The first two genes of the operon 2614, *fliY* (X276_04065) and *fliM* (X276_04060) were responsible for the switch of the flagellar motor and their transcription, as well as those of Che proteins genes (operon 2615), were downregulated between time-points T3 and T4, see Suppl. File 5.

#### Binary fission and asymmetric division

To accomplish division of rod shaped cells, proper assembly of a divisome structure in the middle of the elongating cell is an essential step. The main scaffold for the formation of this division mechanism is provided by FtsZ – a tubulin like protein that forms a Z-ring in the position of the future division septum^72^. To ensure symmetrical division, a group of Min proteins represses the divisome assembly asymmetrically or prematurely. In *E*. *coli*, MinE protein was described to have a dynamic structure, being responsible for MinCD tracking and oscillating from pole to pole^73^, providing dynamic changes in the concentration of the division inhibitor MinCD along the cell, thus inhibiting incorrect positioning of the Z-ring. This oscillatory behaviour of MinE protein was described as being inconsistent with the needs of asymmetric septa formation during sporulation. In *B*. *subtilis*, MinJ and DivIVA carry out the function of MinE to properly localize the MinCD complex through an alternative oscillation-avoiding mechanism^74^.

Most of the essential genes coding for Fts family proteins, the Min group of FtsZ assembly inhibitors and rod shape determining proteins referred to as Mre, were located in the genome and analysed for their expression (see Fig. 6 while RPKM values, putative operon organization and differential expression analysis are shown in Suppl. File 6). Whereas the FtsZ coding sequence was present only once in the genome with a higher expression at the growth stage, a reasonable RPKM value (500–1000) was demonstrated over the entire monitoring period; a number of divisome related genes had more paralogues from which some were active during cell division and others during sporulation. An example is the putative *ftsW*/*ftsI* genes coding for the protein complex that cooperates on synthesis of peptidoglycan in the newly synthesized septa. Both pairs were clustered and putatively regulated together, whereas the X276_01330-800 cluster was upregulated during intensive growth and X276_18745-40 participates in spore coat synthesis and is transcribed more after onset of sporulation (Fig. 7). Close to the X276_01330-800 another *fts* family genes were found, namely *ftsX* (X276_01295) and *ftsE* (X276_01290), which have opposite functions and together regulate cell wall hydrolysis^72^. *FtsA* and *ftsZ* were predicted to form a bi-cistronic operon in *C*. *acetobutylicum*^75^ but even though they were adjacent in *C*. *beijerinckii* (X276_20940, X276_20935) they were apparently regulated differently. The *ftsZ* had a high expression level with a peak at T2 and *fts*A at T4 (see Fig. 6).

Solventogenic clostridia are pleomorphic cells whose morphological changes are connected to metabolic switches and the onset of sporulation^76^. Moreover, cell elongation was observed^77,78^ together with cell degeneration or acid stress. The proper assembly of functional machinery of the bacterial divisome and its regulation significantly influences these transformations. The *fts* genes play a central role in cell division and sporulation and this is probably the reason that their expression was altered in degenerated cells^77^ and genetically transformed cells^63^ that have diverse growth and production parameters. Surprisingly, Tomas *et al*. (2003b) observed upregulation of *ftsA*, *ftsX* and *ftsK* in slower growing cells containing a reference plasmid compared to the wild type *C*. *acetobutylicum*^63^. In contrast, a different study showed that in elongated degenerated *C*. *beijerinckii* NCIMB 8052 cells, *ftsK*, *ftsA* and *ftsY* were downregulated in comparison to shorter CaCO_3_ treated cells; *ftsZ* expression was unchanged^77^.

MreBCD and MinCDE coding genes were co-located in the genome but were not regulated together. According to Genome2D analysis, *MreBCD* (X276_24195 – 55) are part of a predicted wider operon together with three more genes, however from transcriptional analysis, expression of only three *mreBCD* genes correlated together, see Suppl. File 6. Genes for MinCDE (X276_24170 – 80) should form a complete operon^12^ and their transcriptional profile was more or less consistent with this prediction. The *min* and *mre* genes expression was high at the first two measured time-points, where cell division was the most intense. As well as Fts family proteins, some of the *mre* and *min* genes have paralogues within the genome but these were found to be adjacent to sporulation or flagella formation genes and therefore were not considered to play a pivotal role in binary fission.

With respect to the oscillatory features of MinE and its incompatibility with asymmetric division, a recent study by Valencikova *et al*. (2018) indicated that clostridia encodes orthologues of both *B*. *subtilis* (MinJ) and *E*. *coli* (MinE) proteins and that they evolved their own mechanism of Min and DivIVA interactions^79^. Whereas we have not found a *minJ* homologue in the *C*. *beijerinckii* NRRL B598 genome, the orthologue of gene *divIVA* (X276_18695) was present in one copy and was actively transcribed during population growth and initiation of spore formation (see Fig. 6 and Suppl. File 6). DivIVA is a multifunctional protein localized at the cell poles due to its affinity for membrane curvature^80^ and also plays an important role in bacterial sporulation^81^. Its temporal complex with SpoIIE relocates the Z-ring from its mid-cell position to a new polar septa site and ensures asymmetric septa formation^82^.

#### Sporulation

Figure 7A shows schematically the relationship between sporulation as a part of the cell cycle, activities of individual sigma factors, approximate sampling times and putative pathways for granulose synthesis/degradation and dipicolinic acid synthesis in *C*. *beijerinckii* NRRL B-598. It also displays selected genes or groups of genes that were included in the transcriptional analysis (Fig. 7B) and which were grouped based on their potential joint regulation or timely expressions. Putative operon organization of the genes, their RPKM values and analysis of differential expressions are shown in Suppl. File 7.

Granulose metabolism: Usually, sporulation, both in bacilli and clostridia, is divided into 7 stages. From the morphological point of view, sporulation in most clostridia is coupled with typical cell thickening caused by granulose accumulation. Granulose is a reserve glycogen-like polysaccharide composed of D-glucose subunits linked mostly with α-1,4 bonds, which is synthesized in cells of most solventogenic clostridia prior to sporulation and is hydrolysed during sporulation. Granulose synthesis has only been studied in *C*. *pasteurianum* and *C*. *acetobutylicum*, but never in *C*. *beijerinckii* and it seems that genes involved in granulose synthesis/degradation, *glg* and *amyP*, in *C*. *acetobutylicum*^83,84^) lack orthologues in *C*. *beijerinckii*.

Morphology of the pre-sporulating *C*. *beijerinckii* NRRL B-598 cells was tightly associated with cell thickening caused by granulose (glycogen-like polysaccharide) accumulation (see Fig. 1D). The putative granulose synthesis/degradation operons (2952, 2953, see Suppl. File 7) were found in *C*. *beijerinckii* NRRL B-598, and included genes *glgD* (X276_01040), glucose 1-phosphate adenyl transferase (X276_01035), glycoside hydrolase (X276_01030), *glgP* (X276_01025), *glgA* (X276_01020) and *glgB* (X276_01015). A further glucose phosphomutase gene, *pgm* (X276_21930) was also found. A similar organization of granulose-involved genes was found in *C*. *beijerinckii* NCIMB 8052. Liu *et al*.^79^ described transcription of granulose-involved genes in *C*. *acetobutylicum* ATCC 824 but no similarity either in operon organization or in individual genes homologies was found with *C*. *beijerinckii* strains. A probable mechanism of granulose formation in *C*. *beijerinckii* NRRL B-598 (see Fig. 7A) starts with glucose-6-phosphate, which is phosphorylated by PTS transport proteins and includes the following steps: formation of glucose-1-phosphate from glucose -6-phosphate catalysed by phospho glucose mutase (gene X276_21930), activation of glucose-1-phosphate with ATP resulting in ADP-glucose catalysed by glucose-1-phosphate adenyl transferase, *glgC*, *glgD* (genes X276_01035, X276_01040), then α-1,4-glucan synthesis is catalysed by granulose synthase, *glgA* (X276_01020) and the resulting granulose polymer is finished probably by the activity of a branching enzyme coded by *glgB* (X276_01015). Based on the transcription profile of *glgB*, it seems possible that the granulose of *C*. *beijerinckii* NRRL B-598 is not a linear but a branched polymer, also containing α-1,6- glucose bonds. Granulose decomposition might be mediated by *glgP* (X276_01025) through phosphorolysis and/or glycoside hydrolase, *amy* gene (X276_01030), by hydrolysis. Structures of glycogen synthases, both in prokaryotic and eukaryotic kingdoms, exhibit great diversity and can only be compared by function not by structure; only a few of them have been studied in detail. A similar putative glycogen formation/degradation cluster was described in *Lactobacillus mucosae*^85^.

Quorum sensing Agr systems: In *C*. *acetobutylicum* ATCC 824, sporulation and granulose formation might be under the control of the Agr quorum sensing system^86^. A probable model of signal transduction and Agr governed regulation was constructed based on the information gathered for the strain^87^. The Agr quorum sensing system was thoroughly studied in *Staphylococcus aureus*^88^ and typically includes genes for the following proteins: autoinducer signalling peptide (*agrD*), membrane processing and secretion protein (*agrB*), membrane sensor histidine kinase (*agrC*) and transcriptional activator (*agrA*). The Agr system is based on two component regulation, where the autoinducer peptide is post-translationally processed and secreted from cells by AgrB, and then the membrane sensor histidine kinase (AgrC) accepts the signal in the form of the autoinducer peptide and phosphorylates a transcriptional regulator AgrA. Phosphorylated AgrA further interacts with the promoter region of RNAIII, a small regulatory RNA.

Surprisingly, three transcribed gene clusters for Agr quorum sensing systems were found in *C*. *beijerinckii* NRRL B-598 (see Fig. 7B). While two of them seem to contain all four *agrA*, *agrB*, *agrC* and *agrD* putative genes, the remaining one lacked the gene for cyclic autoinducer peptide production (*agrD*). These systems did not share any homology with those found in *C*. *acetobutylicum* ATCC 824, but as in *C*. *acetobutylicum* ATCC 824^86^, the small regulatory RNAIII, transcription of which should be regulated by AgrA-P, was not found. For both *C*. *difficile*^89^ and *C*. *acetobutylicum*^86^ it is supposed that the Agr system might be involved in regulation of phosphorylation of Spo0A. Based on timely expression of all three Agr systems in *C*. *beijerinckii* NRRL B-598, it might also be the case but experimental proof is necessary because not only Agr systems but also histidine kinases potentially involved in Spo0A phosphorylation and thus sporulation initiation were not similar to those found for *C*. *acetobutylicum* ATCC 824.

Sporulation initiation: A comparison of sporulation in different clostridia was provided by Al-Hinai *et al*.^90^. Unlike in bacilli, where the sporulation signal (starvation) is recognized by histidine kinases and transmitted by phosphoryl transferases to sigma factors and sporulation specific regulators, in clostridia, neither the sporulation signal, nor its recognition and transmission have been fully clarified even if some models have already been published^90–92^. The sporulation sequence is usually described until sporulation stage IV-V because late sporulation events remain hidden.

All genes coding main sigma factors involved in sporulation, i.e. *sigH*, *sigF*, *sigE*, *sigG* and *sigK*, were found and their transcriptions (Fig. 7B) fitted well with standard *Clostridium* sporulation models. During vegetative growth (samples T1 and T2), transcription of DNA to RNA is governed by *sigA (sig70)* in prokaryotes. In the strain *C*. *beijerinckii* NRRL B-598, the transcription of *sigA* (X276_22455) was pronounced in T1 but also in T4 (see Fig. 7B). The transcriptional increase at T4 might correspond with a better physiological state of the culture, as confirmed by flow cytometric analysis (see Fig. 1). However, it is also noteworthy that regarding RPKM values (see Suppl. File 7), *sigA* transcription can be considered almost constitutive, which is in accordance with expectation because in all samples, there were always some vegetative cells. In *C*. *acetobutylicum* ATCC 824, *sigA* gene is part ofthe *dnaE-sigA* operon^93^, however in our strain, it was probably part of the operon with *dnaG* coding DNA primase (X276_22460), see Suppl. File 7, as in *C*. *beijerinckii* NCIMB 8052^11^.

For *C*. *acetobutylicum* ATCC 824, there were identified three orphan histidine kinases (ohk) (CA_C0323, CA_C0903 and CA_C3319) that might be involved in direct phosphorylation of Spo0A, and another one (CA_C0437) probably with dephosphorylation activity^90^. Several genes encoding potential histidine kinases were found in *C*. *beijerinckii* NRRL B-598, two of them (X276_26695 and X276_12860) sharing partial homologies with CA_C0323. Especially the first one (X276_26695) had a promising transcription profile and might be involved in Spo0A phosphorylation. However, to prove or disprove this, further research is necessary as many *ohk* genes were found in the *C*. *beijerinckii* NRRL B-598 genome. To reveal histidine kinases involved in Spo0A phosphorylation in *Clostridium thermocellum*^92^, there was performed a multiple step bioinformatics analysis which resulted in 19 potential *ohk* genes selected from 41 original candidates. These genes were knocked-out or over-expressed to identify 4 histidine kinases involved in sporulation. Wang *et al*. (2013) hypothesized direct phosphorylation of Spo0A by butyryl-phosphate in *C*. *beijerinckii* NCIMB 8052^94^, which was also presented by Paredes *et al*. (2005) for *C*. *acetobutylicum* ATCC 824^95^. However, demonstration of Spo0A phosphorylation *in vitro* by these histidine kinases in *C*. *acetobutylicum* ATCC 824 provided evidence that direct phosphorylation of Spo0A by orphan histidine kinases is possible^96^.

Both *spo0A* and *sigH* genes were expressed constitutively at high levels (see RPKM values in Suppl. File 7) although expression of the former gene was higher than expression of the latter. This is in complete accordance with findings for *C*. *acetobutylicum* ATCC 824 and *C*. *beijerinckii* NCIMB 8052^12,90^. In bacilli and in *C*. *acetobutylicum* ATCC 824, *sigH* expression in vegetative cells was repressed by AbrB and derepression occurred under excess of Spo0A-P. In *C*. *beijerinckii* NRRL B-598, a potential *AbrB* gene (X276_01205) exhibiting a corresponding expression profile was identified. In *C*. *beijerinckii* NCIMB 8052^12^, several genes potentially coding for AbrB proteins were found, one of them (Cbei_4885) sharing homology with (X276_01205) but without a matching expression profile.

Sporulation phases from I to IV/V: Most key sporulation steps are common for both clostridia and bacilli^97^ but for the model solventogenic strain *C*. *acetobutylicum* ATCC 824, early sporulation events were described the best by Durre (2014) and Al-Hinai *et al*.^90,91^. Based on known models, corresponding genes were searched for *C*. *beijerinckii* NRRL B-598 and a sequence of sporulation events based on transcription and genome data (see Fig. 7A,B) was reconstructed. The early sporulation sequence might proceed in the following way: in the forespore, sigma F factor (gene X276_22650) is active when unphosphorylated anti-sigma factor SpoIIAB (gene X276_22655) binds to unphosphorylated anti-anti-sigma factor SpoIIAA (gene X276_22660) while in the mother cell, sigma F is inactive due to its bond with phosphorylated SpoIIAA-P; SpoIIAB-P can bind to sigma F but can also phosphorylate SpoIIAA while SpoIIE (gene X276_26240) mediates dephosphorylation; sigma F upregulates expression of *spoIIR* (gene X276_24685) and the corresponding protein binds with membrane bound SpoIIGA (gene X276_20930); SpoIIGA hydrolyzes pro-sigma E in the mother cell to active sigma E (gene X276_20925); the joint expression regulation of other genes by sigma F and sigma E in the forespore and mother cell, respectively, results in engulfment of the pre-spore; this is usually referred as sporulation stage II. In agreement with findings for *C*. *acetobutylicum* ATCC 824^98^, *spoIIGA-sigE-sigG* were also clustered together and probably form an operon together with *ylmC* (X276_20915) and *nrdR* (X276_20910) genes (see operon 0621 in Suppl. File 7) and, in a similar way upstream of this operon, *ftsZ* and *ftsA* involved in cell division were located. It was proven experimentally in *C*. *acetobutylicum* ATCC 824^99^, that the gene *spoIIE* was responsible for the correct position of the forespore septum in the mother cell through its interaction with the *ftsZ* gene. In the putative *spoIIGA-sigE-sigG* operon (no. 0621) was located gene *nrdR* (X276_20910), a known bacterial transcriptional repressor of ribonucleotide reductase, which is involved in cell division and DNA repair. Thus, it cannot be excluded that NrdR functions as a putative repressor of standard cell division in sporulating cells. This hypothesis fits with its transcriptomic profile but requires further study. There were other genes found, probably responsible for the engulfment process of *C*. *beijerinckii* NRRL B-598, and included the SpoIIIA complex in the mother cell, containing 8 adjacent genes (from X276_18580 to X276_18545) and *spoIIQ* (X276_24540), *spoIID* (X276_24545), *spoIIM* (X276_17010) and *spoIIP* (X276_22605) in the forespore. The coordinated action of spoIIM, spoIIP and spoIID proteins, essential for the correct engulfment process was described for *B*. *anthracis* and *Clostridium difficile*^100^. SpoIIQ and SpoIIIA complexes were demonstrated to be involved in spore septum formation and function and correct localization of sigma factor activities in *C*. *difficile*^101^.

Sigma G (gene X276_20920) and sigma K (gene X276_20950) factors then become active in the forespore and mother cell, respectively. Genes probably involved in sigK formation i.e. sigK transcriptional regulator *spoIIID* (X276_24535) and genes involved in pro-sigK processing, *spoIVFB* (X276_24145) and *yunB* (X276_04420) were identified. Furthermore, *spoVG* (X276_26375) transcription was at a high level from the beginning of cultivation until sampling at T5, which agrees with the expectation that SpoVG protein acts as a negative regulator of asymmetric division^102^. High levels of *spoVG* transcription at all time-points implies its probable more complex role in the physiology of the tested organism. During tests with *Listeria monocytogenes*, a nonsporulating G^+^ bacteria, it was concluded that SpoVG might be a global posttranscriptional regulator conserved in many bacteria^103^.

Further spore development (i.e. from sporulation stage III/IV) differs from bacilli significantly and has never been described in detail in solventogenic clostridia, not even for *C*. *acetobutylicum* ATCC 824. Late sporulation events include formation of the spore core, spore cortex and spore coat, and eventually, exosporium development. Spore coat protein formation probably starts in sporulation stage IV. In *B*. *subtilis* CotE, SpoIVA and SafA proteins are so-called morphogenetic and their deletions resulted in incomplete spore development^104,105^. Only the *spoIVA* orthologue (X276_20845) was identified in our genome. In addition, some spore coat protein genes, namely *cotS (*X276_24750, X276_24745, X276_24740, X276_24730, X276_22470), *cotJA (*X276_16495), *cotJB (*X276_16500), *cotJC (*X276_16505), and *cotF* (X276_19150, X276_19140, X276_19135), which should code for proteins forming inner and outer layers of the spore coat, were identified. For both bacilli and clostridia, it is known that some spore coat proteins are classified as manganese catalases, superoxide dismutases or oxidases. It is assumed that they might play a protective role to prevent oxidative shock, especially during germination of spores because they usually do not increase resistance of spores against hydrogen peroxide or similar chemicals. In *C*. *beijerinckii* NRRL B-598, CotJC seems to have the function of a manganese catalase.

Furthermore, nine genes for small acid-soluble spore proteins (SSPs) that stabilize DNA in the spore core were identified. Germination protein GerA (gene X276_21680) plus *spoVAC* (X276_22645), *spoVAD* (X276_18765) and *spoVAE* (X276_22635) genes, whose protein products might function in mature spores as channels enabling specific low molecular weight molecules to overcome spore coat layers and to start germination^106^, were identified as well. *Ssp*, g*erA* and spore channel genes exhibited increased transcription at time-points T4-T6, which corresponded well with expected spore development. DNA in the spore core is stabilized not only by small acid soluble proteins but also by calcium dipicolinate. A putative mechanism for dipicolinate (DPA) formation in *C*. *beijerinckii* NRRL B-598 was reconstructed from genomic and transcriptomic data and its scheme is shown in Fig. 7A. Dipicolinate formation is part of the lysine biosynthetic pathway and includes formation of semialdehyde aspartate from L-aspartyl-4-P, *asd* gene (X276_18050), followed by condensation of semialdehyde aspartate with pyruvate catalysed by 4-hydroxy-tetrahydropicolinate synthase, *dapA* genes (X276_18055, X276_01925), resulting in the formation of 4-hydroxy-tetradipicolinate (HTPA). This may be spontaneously or enzymatically (by 4-hydroxy-tetrahydrodipicolinate reductase, *dapB*, gene X276_18060) dehydrated to dihydrodipicolinate (DHDPA). We identified two *dapA* genes, one in the putative operon with *dapB* and *asd* (operon 0922, see Suppl. File 7) and the second, putatively monocistronic. Both were transcribed during the whole fermentation, implying that both contribute to L-lysine synthesis. However, there was increased transcription of *dapA*, *dapB* genes organized in the operon 0922 in samples from time-points T5–T6, which correlates with an increased need in sporulating cells for DPA synthesis. The last oxidation step of dipicolinate synthesis from DHDPA to DPA is mediated by a protein encoded by *spoVF* in bacilli. However, no orthologue of this gene was found in the *C*. *beijerinckii* NRRL B-598 genome. Thus, it is possible that oxidation is performed by electron transfer flavoprotein subunit alpha (EtfA), as in *C*. *perfringens*^107^. We identified four Etf systems, all of which had putative heterodimer structures, composed of Etf subunits alpha and beta. The Etf system (X276_25275, X276_25270) that might be involved in DHDPA oxidation was chosen based on increased transcription observed during the T4 – T6 time interval, see Fig. 7B.

In addition to SSPs and dipicolinic acid, germination proteins are also found in the spore core or create a part of the inner spore membrane. From this group of proteins, we identified *gerA* (X276_21680), germination receptor, genes coding lytic enzymes, *yyaC* (X276_00070), *gpr* (X276_22610) and spore cortex lytic protein (X276_09915). We also tracked the *spoVA* operon (operon 0435, see Suppl. File 7), putatively involved in formation of mechano-sensitive channels necessary for DPA release and water uptake during germination. The important role of SpoVA proteins in germination was described recently for *C*. *difficile*^108^.

Lastly, *spoVD* and *spoVE* operons (operons 0853-0855; adjacent genes X276_18780- X276_18740; see Fig. 7 and Suppl. File 7) putatively involved in spore cortex formation were identified. Part of operon 0853 is the *mraZ* repressor (X276_18780), putatively involved in regulation of *spoVD* and *spoVE* operons; this was massively transcribed from time-point T4 in accord with spore cortex formation. The operon contains genes for peptidoglycan synthesis *murE (*X276_18755), *murF (*X276_18750), and *mraY (*X276_18745). A similar organization of cortex formation genes was described in *B*. *subtilis*^109^. Very probably, other genes are involved in cortex formation but as they are usually also involved in other cellular functions in vegetative cells, such as cell wall genesis/remodelling, during acid/solvent formation, they are difficult to find.

### Concluding remarks

The organization of genes in the genome of *C*. *beijerinckii* NRRL B-598, as well as their transcription, reflect long years of evolution. This extensive study has given us a deeper insight into the *C*. *beijerinckii* life cycle and reveals new possibilities for strain improvement. It also helped to confirm or disprove some hypotheses and has revealed expression interrelationships of selected genes, both in and out of the predicted operons.

Central glucose catabolism was mapped in detail for *C*. *beijerinckii* NRRL B-598. In most features, the strain was very similar to *C*. *beijerinckii* NCIMB 8052. The expression profile followed the growth and production characteristics with unambiguous upregulation of solventogenesis-related genes at the pH breakpoint. With regard to the most extensive change in expression of individual genes recorded at later stages, it was demonstrated that the acidogenesis and solventogenesis switch might not be the main event in the clostridial lifecycle and from the transcriptomic profiling data, solventogenesis and sporulation might be independent events.

Alteration of unsaturated fatty acids with cyclopropanated ones correlated with butanol production. This brought further indirect evidence that stabilization and increased fluidity of cellular membranes might be achieved by this mechanism, which seems to be an important stress response to butanol formation. Heat shock protein production reflected both acid and solvent formation. While class I HSPs are probably involved in overcoming low pH stress, upregulation of specific stress-related sigma factor I together with class II, III and unclassified *hsp* genes might be caused by butanol production and accumulation.

Putative pathways for granulose and dipicolinic acid formation, which have never been studied in *C*. *beijerinckii*, were described. Based on the transcription of granulose formation genes it cannot be excluded that *C*. *beijerinckii* granulose might be a branched rather than linear glucose polymer. While dipicolinic acid formation originates from the L-lysine biosynthetic pathway, specific genes *dapA*, *dapB*, which probably strengthen production of dipicolinic acid during sporulation, were identified. Three actively transcribed clusters of genes for Agr quorum sensing were identified. However, it can be only hypothesized as to which cellular or metabolic processes they might regulate. They might be involved in granulose formation but also in sporulation or solventogenesis. - Both an actual role of the Spo0A regulator and a mechanism for its phosphorylation remain unknown. While it seems clear that it is involved in the initiation of sporulation, its engagement in the solventogenic switch and its potential negative regulation of chemotaxis/motility is still an open question.

## Material and Methods

### Bacterial strain and culture conditions

*C*. *beijerinckii* NRRL B-598 (ARS/NRRL culture collection) was stored in the form of spore suspensions in sterile distilled water in a refrigerator at 4 °C.

The inoculum for batch fermentation experiments was prepared from the spore suspension of the strain. After heat treatment at 80 °C for 2 min, the suspension was transferred to modified TYA broth^9^ containing 20 g/L of glucose and the inoculum was cultivated overnight under a stable 90% N_2_, 10% H_2_ atmosphere (Concept 400 anaerobic chamber, Ruskinn).

The bacterial strain was cultivated in parallel Multifors 1L bioreactors (Infors HT) at 37 °C in respective culture broths with a glucose concentration of 50 g/L. Prior to inoculation, oxygen was removed from the reactors by N_2_ washing and the pH of the culture broth was adjusted to 6.3. Henceforth pH was measured but not controlled. Each bioreactor contained 630 mL of medium and was inoculated with 70 mL of cell culture.

### Characterization of cell growth, morphology and physiology

Cell dry weight of biomass (CDW) was used for the determination of cell concentration after drying until constant weight at 105 °C. All samples were analysed in three repetitions.

Cell morphology was observed by phase contrast microscopy (Olympus BX51 microscope) with ×400 and ×1000 magnifications. Photographs of cells were taken using an EOS 600D camera (Canon).

Cell culture viability and the amount of spores formed were determined using flow cytometry (BD Accuri C6). Combined propidium iodide PI (Sigma Aldrich) and carboxy fluorescein diacetate CFDA (Sigma Aldrich) fluorescent staining was applied prior to the measurement. All experiments were carried out according to the method established by Branska *et al*.^14^.

### Quantification of substrate consumption and metabolite production

HPLC with refractive index detection (Agilent Series 1200 HPLC; Agilent), in combination with IEX H + polymer column (Watrex) were used for the determination of glucose consumption and production of metabolites: butanol, acetone, ethanol, lactic acid, acetic acid, and butyric acid. Samples were prepared from microfiltered supernatants of cell broths and conditions of analysis were as described previously^9^.

### RNA extraction and sequencing

In this study, we combined two biological replicates covering six samples each (B1–B6, C1–C6) from our previous study^10^ with corresponding newly sequenced technical replicates (D1–D6, E1–E6). Therefore, the whole dataset consisted of 24 samples covering six time-points (T1–T6) during cultivation by four replicates each (B, C, D, E).

New technical replicates presented in this study were obtained by re-sequencing of samples presented in our previous study^10^. For RNA extraction, cell samples were taken from each bioreactor at six time-points (3.5, 6, 8.5, 13, 18, and 23 h of cultivation) so that acidogenic and solventogenic phases of growth, as well as the sporulation cycle, were covered. The sampling time 3.5 h (T1) corresponds to acids but not butanol production, sampling point 6 h (T2) corresponds with the lowest pH reached and the start time of solventogenesis while sampling point 8.5 h (T3) reflects acids re-utilization and solvent formation. At sampling time 13 h (T4), increased number of viable cells was observed in the population along with the accumulation of granulose in cells and the probable start of sporulation. Sampling times 18 h (T5) and 23 h (T6) reflect progressive sporulation together with solvents accumulation in the culture medium. Irregular sampling was chosen as best fit for the main life cycle and metabolic changes of the culture. Cell samples were prepared from 3 ml of culture broth at OD_600_ = 0.9–1.0 and stored at −70 °C. Total RNA was extracted using a High Pure RNA Isolation Kit (Roche), and 16S and 23S ribosomal RNAs were removed using The MICROB*Express*™ Bacterial mRNA Enrichment Kit (Ambion). RNA samples were stored at −70 °C.

An Agilent 2100 Bioanalyzer (Agilent) with the Agilent RNA 6000 Nano Kit (Agilent) and DS-11 FX + Spectrophotometer (DeNovix) were used for quality control and concentration measurements of RNA samples.

Library construction and sequencing of samples from technical replicates were performed by the CEITEC Genomics core facility (Brno, Czechia) on Illumina NextSeq500, single-end, 75 bp.

### Bioinformatics analysis

Data pre-processing of new replicates (D, E) was performed in the same manner as in our previous study^10^. FastQC in combination with MultiQC^110^ were used for quality assessment of particular steps, including filtering out 16S and 23S rRNA contamination with SortMeRNA^111^ utilizing the SILVA database^112^ and mapping of cleansed reads to the reference genome of *C*. *beijerinckii* NRRL B-598 (NZ_CP011966.2) with STAR^113^. Resulting SAM (Sequence Read Alignment/Map) files were indexed and transformed into more compact the BAM (Binary Read Alignment/Map) format using SAMtools^114^.

The count table from all 24 samples was reconstructed using the R/Bioconductor featureCounts function from the Rsubread package^115^. The contribution of multi-mapping reads caused by gene duplications was down-weighted by the number of times these reads mapped to the genome. Similarly, reads mapping to more genomic objects were down-weighted by the number of genomics objects they mapped to. The data normalization step that is important for the most accurate comparison of transcription due to differences between libraries, varying sample-to-sample and differences in the read coverage, was performed with the R/Bioconductor DESeq2 package^116^. Raw count table data were normalized using a built-in DESeq2 function that deals with differences both in library sizes and composition. Visualization of normalized samples after dimensionality reduction was created with Rtsne using Barnes-Hut t-SNE implementation^117^ and ggplot2^118^ R packages. Transcription profiles of selected genes were visualized as heatmaps of Z-scores with R packages, gplots and RColorBrewer. RPKM values were calculated using the R/Bioconductor edgeR package^119^ as auxiliary information regarding expression of genes. Operon structure predicted by Genome2D^120^, available as a webserver application (version 2.0, July 2018) at http://genome2d.molgenrug.nl/index.html was verified using the Pearson correlation of transcription profiles of particular genes within operons. Time series and bar plots were generated with Matlab 2017b.

## Supplementary information

RNA-Seq data statistics

Dataset 2

Dataset 2a

Dataset 3

Dataset 4

Dataset 5

Dataset 5a

Dataset 6

Dataset 7

## Data Availability

The genome assembly referred to in this paper is version NZ_CP011966.2, obtained from the NCBI RefSeq database. The RNA-Seq sequencing data are available from the NCBI Sequence Read Archive (SRA) under the accession number SRP033480 that includes novel technical replicates published within this study under accession numbers SRX4501422 and SRX4501423.
