# Supplementary material for: Acidogenesis, solventogenesis, metabolic stress response and life cycle changes in Clostridium beijerinckii NRRL B-598 at the transcriptomic level
Source: Sci Rep. 2019 Feb 4;9:1371. doi: 10.1038/s41598-018-37679-0 (PMC6362236; doi:10.1038/s41598-018-37679-0)

**Acidogenesis, solventogenesis, metabolic stress response and life cycle changes in *Clostridium beijerinckii* NRRL B-598 at the transcriptomic level**

Petra Patakova^1*^, Barbora Branska^1^, Karel Sedlar^2^, Maryna Vasylkivska^1^, Katerina Jureckova^2^, Jan Kolek^1^, Pavlina Koscova^2^, Ivo Provaznik^2^

^1^Department of Biotechnology, University of Chemistry and Technology Prague, Technicka 5, 166 28 Prague, Czech Republic.

^2^Department of Biomedical Engineering, Brno University of Technology, Technicka 12, 616 00 Brno, Czech Republic.

*corresponding author, e-mail:petra.patakova@vscht.cz


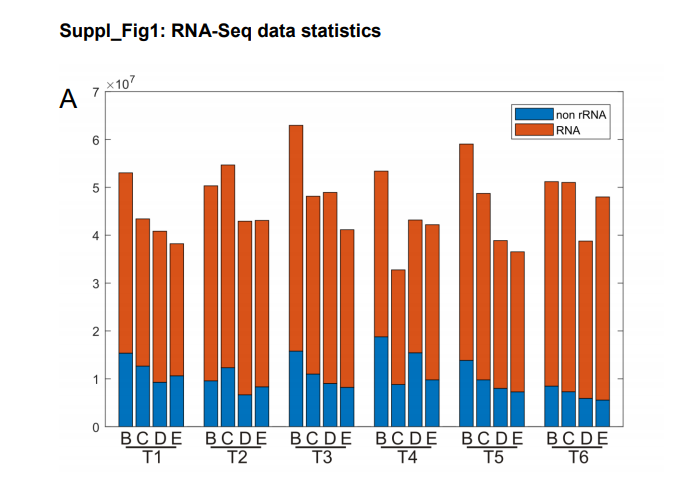


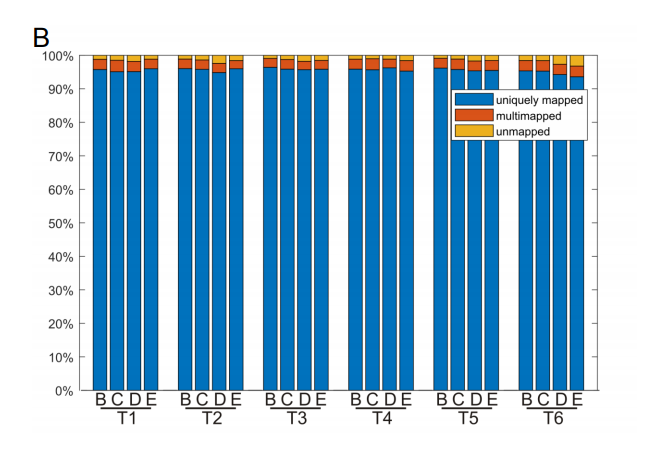


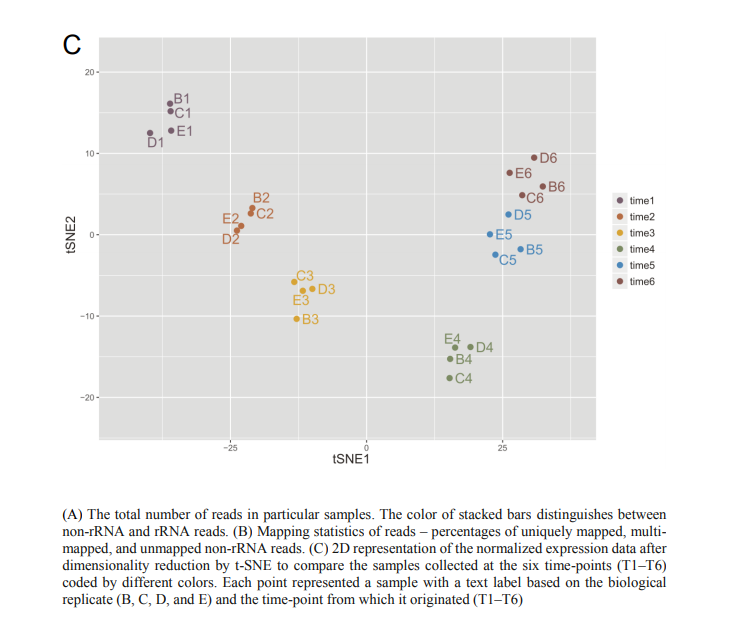

Supplement: Supplementary file 1 — RNA-Seq data statistics [file 41598_2018_37679_MOESM1_ESM.docx]
